# The Impact of Disease Comorbidities in Alzheimer's Disease

**DOI:** 10.3389/fnagi.2021.631770

**Published:** 2021-02-12

**Authors:** Jose A. Santiago, Judith A. Potashkin

**Affiliations:** ^1^NeuroHub Analytics, LLC, Chicago, IL, United States; ^2^Cellular and Molecular Pharmacology Department, Center for Neurodegenerative Diseases and Therapeutics, The Chicago Medical School, Rosalind Franklin University of Medicine and Science, North Chicago, IL, United States

**Keywords:** Alzheimer's disease, cardiovascular disease, comorbidities, depression, gut microbiome, inflammation

## Abstract

A wide range of comorbid diseases is associated with Alzheimer's disease (AD), the most common neurodegenerative disease worldwide. Evidence from clinical and molecular studies suggest that chronic diseases, including diabetes, cardiovascular disease, depression, and inflammatory bowel disease, may be associated with an increased risk of AD in different populations. Disruption in several shared biological pathways has been proposed as the underlying mechanism for the association between AD and these comorbidities. Notably, inflammation is a common dysregulated pathway shared by most of the comorbidities associated with AD. Some drugs commonly prescribed to patients with diabetes and cardiovascular disease have shown promising results in AD patients. Systems-based biology studies have identified common genetic factors and dysregulated pathways that may explain the relationship of comorbid disorders in AD. Nonetheless, the precise mechanisms for the occurrence of disease comorbidities in AD are not entirely understood. Here, we discuss the impact of the most common comorbidities in the clinical management of AD patients.

## Introduction

Alzheimer's disease (AD) is the most common neurodegenerative disease affecting around 50 million people worldwide (Alzheimer's Association, 2016). Accumulation of extracellular amyloid beta plaques and intraneuronal neurofibrillary tangles are hallmark features of the disease (Bloom, 2014). Although several causative genetic factors have been identified, the vast majority of the cases are sporadic. Indeed, environmental factors and lifestyle choices appear to be the main determinants of the disease.

**Figure 1:**
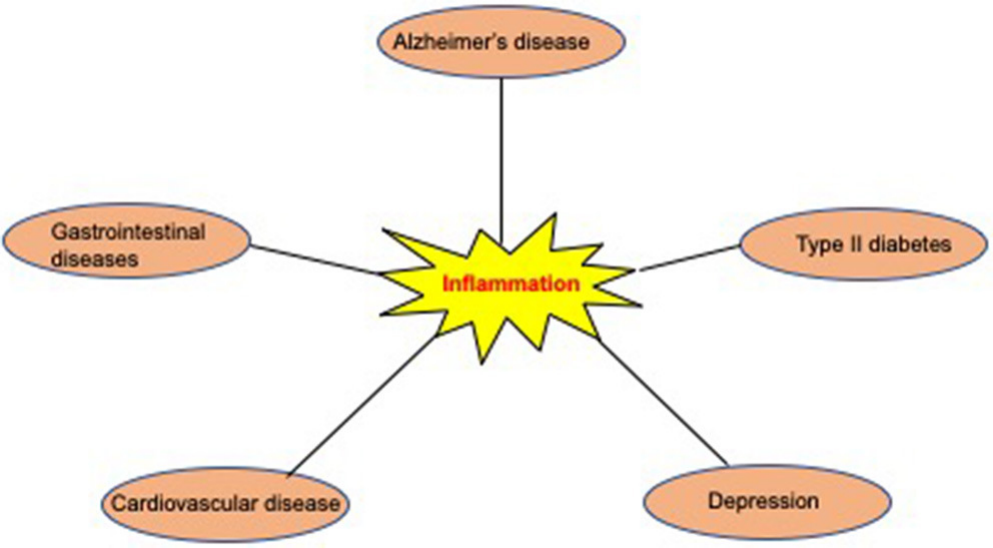
Most common comorbidities associated with AD. Evidence from epidemiological and molecular studies suggest that several conditions including type 2 diabetes, cardiovascular diseases, depression, and gastrointestinal diseases may be associated with an increased risk for AD. Inflammation may be a central mechanism underlying the association between AD and most of its comorbidities.

For several decades, AD patients have been classified according to several clinical measurement scales that primarily determine cognitive impairment status in patients. AD patients are staged into three main clinical categories that include pre-clinical AD, mild cognitive impairment (MCI), and overt AD (Albert et al., 2011). The current classification system does not consider important disease prognostic factors, such as the presence of coexisting disease conditions. Comorbid diseases may occur before or concomitantly with AD and may affect the disease's overall clinical status and progression. Several lines of evidence have established associations between AD and other chronic diseases, including diabetes, cardiovascular disease, depression, and inflammatory bowel disease (Casserly and Topol, 2004; Chatterjee and Mudher, 2018) (Ownby et al., 2006; Zhou et al., 2015) (Fu et al., 2020) (Figure 1). In addition to these diseases, neuropathological investigations have revealed an increasing frequency of overlapping co-pathologies, including co-aggregates of TDP-43 in AD patients' brains that could lead to faster progression and atypical clinical presentation (Matej et al., 2019). The presence of coexisting disease conditions may ultimately have a detrimental impact on AD patients' disease management. Understanding the biological mechanisms leading to comorbid diseases in AD may provide novel routes for therapeutic interventions. To this end, herein, we discuss the most prevalent disease comorbidities in AD and their impact on the clinical management of AD patients.

## Alzheimer's Disease and Diabetes

According to the World Health Organization, type 2 diabetes (T2D) is the most prevalent metabolic disease affecting 422 million people worldwide. Hyperglycemia and insulin resistance are characteristic features of the disease. Numerous lines of evidence support the association between T2D and AD. T2D is a well-established risk factor for AD, and AD is sometimes referred to as diabetes type 3 (Kandimalla et al., 2017; de la Monte, 2019). Substantial evidence from epidemiological studies indicates T2D is associated with an increased risk of AD in several populations. A systematic review of 14 longitudinal studies revealed a high risk of AD and vascular dementia among T2D patients (Biessels et al., 2006). This study suggested that vascular disease complications, alterations in insulin, glucose, and amyloid metabolism may underlie the association between both diseases (Biessels et al., 2006). Another study found a significantly lower cognitive performance among diabetic patients compared to healthy controls after 4 years follow-up period (Fontbonne et al., 2001). This study found a 2–3-fold increase in developing dementia in diabetes patients. Similarly, a cross-sectional study found that subjects with T2D performed worse in all cognitive domains than those with normal glucose metabolism (Geijselaers et al., 2017). Consistent with these findings, a recent meta-analysis of 144 prospective studies identified a 1.25–1.9-fold increase for cognitive impairment and dementia in patients with diabetes (Xue et al., 2019).

Glycated hemoglobin levels are an indicator of diabetes used by most clinicians. In this regard, a study identified significant longitudinal associations between hemoglobin A1c (HbAc1) levels, diabetes status, and accelerated cognitive decline over 10 years of follow-up (Zheng et al., 2018). Patients with prediabetes also displayed an increased risk for dementia, suggesting that even early alterations in glucose metabolism can trigger neurodegeneration. In support of these findings, a population-based cohort study showed that diabetes and prediabetes are associated with accelerated cognitive decline (Marseglia et al., 2019). A prospective cohort study found that diabetes in midlife was associated with more significant cognitive decline over 20 years compared to non-diabetic patients (Rawlings et al., 2014). In another study, 81% of AD patients exhibited impaired fasting glucose and diabetes (Janson et al., 2004), thereby demonstrating the high prevalence of diabetes among AD patients. The same study also identified islet amyloid, a pathological hallmark of diabetes, in AD patients compared to normal subjects. More extensive studies have corroborated these results. For example, a meta-analysis of 19 studies, including over 6,000 subjects with diabetes, showed that individuals with diabetes had a higher risk for AD than healthy controls (Cheng et al., 2012). Contrary to these findings, several studies have reported no association between diabetes and AD (Hassing et al., 2002; MacKnight et al., 2002; Akomolafe et al., 2006). A summary of the main findings of epidemiological studies addressing the relationship between diabetes and AD and cognitive decline is presented in Table 1.

**Table 1:** Epidemiological studies investigating the association between AD, dementia, cognitive impairment, and diabetes.

**Study**	**Study design**	**Main results**
**Alzheimer's disease**		
Janson et al. (2004)	Cohort study	Diabetes or impaired fasting glucose was present in 81% of AD patients
Cheng et al. (2012)	Meta-analysis	Diabetes associated with a higher risk of AD
**Dementia**		
Fontbonne et al. (2001)	Cohort study	Diabetes associated with a 2–3-fold increase risk of dementia
Biessels et al. (2006)	Systematic review	The incidence of dementia was higher in diabetes compared to non-diabetic patients
**Cognitive impairment and dementia**		
Rawlings et al. (2014)	Cohort study	Diabetes in midlife was associated with a 19% greater cognitive decline in 20 years
Geijselaers et al. (2017)	Cross-sectional	Diabetes associated with cognitive decline
Zheng et al. (2018)	Cohort study	Diabetes and HbAc1 levels associated with cognitive decline in 10 years follow up. Prediabetes associated with an increased risk of dementia
Xue et al. (2019)	Meta-analysis	Diabetes associated with a 1.25–1.9-fold increase in cognitive impairment and dementia
Marseglia et al. (2019)	Cohort study	Diabetes and prediabetes associated with accelerated cognitive impairment

Despite the numerous lines of evidence linking T2D and AD, this association's underlying mechanism remains poorly understood. Several mechanisms for this linkage have been postulated, including impaired glucose metabolism, vascular abnormalities, impaired insulin signaling, amyloidosis, and inflammation (Chatterjee and Mudher, 2018). For example, HbAc1, a measure of average blood glucose level, has been positively associated with the increased risk of cognitive decline and dementia in several studies (Yaffe et al., 2006; Rawlings et al., 2017; Zheng et al., 2018). Decreased brain glucose metabolism has been documented in subjects with MCI and T2D compared to those with MCI but not T2D suggesting that T2D may accelerate cognitive impairment (Li W. et al., 2016). In the context of vascular abnormalities, patients with T2D had a higher risk of cerebral amyloid angiopathy (Peila et al., 2002), a condition associated with brain infarcts and AD (Merlini et al., 2016; Noguchi-Shinohara et al., 2017).

Another potential mechanism linking AD and T2D is hyperglycemia. One notable example was illustrated using a murine model of AD wherein the induction of acute hyperglycemia increased amyloid beta in hippocampal interstitial fluid in young animals and prominent amyloid beta plaques in aged mice (Macauley et al., 2015). Nonetheless, more mechanistic studies are needed to determine whether these results can be recapitulated in humans.

Impaired insulin signaling is one of the most supported hypotheses linking T2D with cognitive decline and dementia. Alterations in the phosphatidylinositol 3-kinase and protein kinase B/Akt PI3K-AKT pathway in both T2D and AD patients have been observed by numerous studies suggesting this pathway may play a critical role in the development of AD among T2D patients (Liu et al., 2011; Gabbouj et al., 2019; Santiago et al., 2019). For example, decreased activity of several components of the PI3K-AKT pathway was found in the frontal cortex of both T2D and AD patients postmortem (Liu et al., 2011). Another study identified increased levels of insulin-like growth factor receptor (IGF-1R) and decreased levels of insulin receptor binding protein-2 (IGBP-2) in the temporal cortex of AD patients (Moloney et al., 2010). These findings provided evidence for the presence of insulin resistance in the brain of AD patients.

Amyloidosis is also a common shared pathological feature in T2D and AD. Accumulation of amylin polypeptide in pancreatic islets is present in 95% of T2D patients, and it has been demonstrated to impair islet function (Cooper et al., 1987). Furthermore, both amyloid β and amylin accumulate in tissues in response to innate immune responses or bacterial infections (Miklossy and McGeer, 2016). These findings support the hypothesis that T2D, like AD, may result from a protein misfolding mechanism (Mukherjee et al., 2015).

The immune system has been shown to play a pivotal role in the development of AD and T2D. Increased proinflammatory cytokines in both diseases is one of the most common findings identified in numerous studies. For instance, elevated cytokines and chemokines have been found in T2D (Boni-Schnetzler et al., 2008) and AD patients (Lai et al., 2017). Furthermore, increased levels of peripheral inflammatory markers are associated with disease progression in AD (Italiani et al., 2018). The proinflammatory cytokine tumor necrosis factor (TNF) is known to trigger insulin resistance (Hotamisligil et al., 1993) and exacerbate the accumulation of amyloid beta in AD models (Blasko et al., 1999; Liao et al., 2004). Therefore, targeting TNF signaling is being investigated as a potential therapeutic for AD (Decourt et al., 2017).

Diseases that share common dysregulated pathways are likely to share some of the same therapeutic targets. In this regard, drugs commonly prescribed for treating T2D have shown some promise in AD patients. Antidiabetic medications such as metformin and glucagon-like peptide 1 receptor agonists (GLP-1) have been investigated as potential AD therapies. For example, long-term and high dose metformin use was associated with a lower risk of incident AD in T2D patients (Sluggett et al., 2020). Similarly, a meta-analysis of 14 studies showed the use of metformin was associated with a reduced risk of dementia in T2D patients (Campbell et al., 2018). A small pilot study showed metformin associated with improved executive function, memory, and attention in a group of non-diabetic patients with MCI and AD (Koenig et al., 2017). Contrary to these findings, the potential neuroprotective effect of metformin has been challenged by other investigations. For instance, a population-based case-control study, including more than 7,000 individuals, found that long-term use of metformin associated with a greater risk of developing AD (Imfeld et al., 2012). Likewise, a cohort study including 4,651 elderly patients with T2D found that metformin's long-term usage increased the risk of developing PD, AD, and vascular dementia (Kuan et al., 2017). Therefore, additional larger prospective and randomized controlled trials are required to evaluate metformin as a potential drug for preventing AD.

Another promising group of antidiabetic drugs, the dual glucagon-like peptide and glucose-dependent insulinotropic peptide (GLP-1/GIP) receptor agonists, have shown neuroprotective effects in animal models of AD (Holscher, 2018; Zhang and Holscher, 2020). Exendin-4 (exenatide), a GLP-1 receptor agonist, has been shown to improve motor symptoms in PD clinical trials (Aviles-Olmos et al., 2013; Athauda et al., 2017). Recently, a double-blinded placebo-controlled trial found that exenatide was safe and well-tolerated in AD patients and lowered Aβ42 levels in extracellular vesicles (Mullins et al., 2019). However, exenatide treatment did not produce significant changes in cognitive measures and biomarkers in CSF. Notwithstanding, it is essential to note that the study evaluated a small number of subjects (*N* = 21) from a single center for 18 months. The small sample size and the early termination of the trial may explain the negative outcomes. A randomized placebo-controlled trial with 38 AD patients showed that liraglutide, another GLP-1 agonist, increased the blood-brain glucose transport capacity in the AD treated group compared to placebo (Gejl et al., 2017). This finding is promising in light of the several studies that suggest that reduction in the glucose transporters in the brain and impaired glucose metabolism may be early pathogenic events that exacerbate neurodegeneration in AD (Guo et al., 2005; Liu et al., 2008, 2009; Winkler et al., 2015). Future evaluation in more extensive and well-characterized clinical trials will be valuable to determine their therapeutic potential for AD.

## Alzheimer's Disease and Cardiovascular Disease

Cardiovascular risk factors have long been recognized as closely related to the development of AD. The impact of cardiovascular risk factors in AD has been documented at clinical and pathological levels (Table 2). The first studies that recognized a potential link between cardiovascular disease and AD correlated the presence of brain infarcts with greater cognitive decline and dementia compared to those without brain lesions (Snowdon et al., 1997). Concurrent cerebrovascular disease was documented to be more commonly observed in AD than in other neurodegenerative diseases (Toledo et al., 2013).

**Table 2:** Studies investigating the association between cardiovascular risk factors, dementia and AD.

**Study**	**Study design**	**Main results**
**Alzheimer's disease**		
Petrovitch et al. (2000)	Longitudinal cohort study	Elevated blood pressure in midlife associated with the development of neuritic plaques and neurofibrillary tangles in AD
Khachaturian et al. (2006)	Population-based cohort study	Use of antihypertensive drugs associated with a lower incidence of AD
**Dementia**		
Guo et al. (1999)	Community-based cohort study	Use of antihypertensive drugs associated with a decreased risk for dementia
van Dijk et al. (2004)	Community-based cohort study	Hypertension associated with severe white matter lesions in non-demented individuals
Peila et al. (2006)	Population-based cohort study	Use of antihypertensive drugs associated with a reduced risk for dementia and cognitive decline in men

## Alzheimer's Disease and Stroke

Cardiovascular diseases, including stroke, atrial fibrillation, and coronary heart disease, have been linked to AD. Lacunar strokes, also known as silent brain infarcts, are the most common type of ischemic stroke and results from the occlusion of blood vessels responsible for supplying deep brain structures. Several studies have shown that lacunar strokes greatly increase the risk of cognitive decline and AD. An earlier prospective study showed that the presence of silent brain infarcts at baseline more than doubled the risk of dementia (Vermeer et al., 2003). Similarly, silent brain infarcts are associated with brain atrophy and increased risk of cognitive impairment and dementia (Thong et al., 2013). These findings have been supported by larger studies. For example, a meta-analysis of 7 cohort studies and 2 nested case-control studies showed that stroke increased risk for AD (Zhou et al., 2015). Furthermore, white matter lesions, characteristic of ischemic stroke, are associated with cognitive decline and AD (Prins et al., 2004; Inaba et al., 2011). Another study found that increased fibrinogen associated with a greater increased in dementia in older subjects with white matter lesions (Hainsworth et al., 2017). This study suggested that some degree of blood-brain barrier dysfunction in older people may be related to risk for dementia. The main results from epidemiological studies investigating the association between AD and cardiovascular disease are presented in Table 3.

**Table 3:** Epidemiological studies investigating the association between AD, dementia, cognitive impairment and cardiovascular disease.

**Study**	**Study design**	**Main results**
**Alzheimer's disease**		
Hofman et al. (1997)	Population-based study	Atherosclerosis associated with a higher risk for AD and vascular dementia
Bunch et al. (2010)	Prospective cohort study	Atrial fibrillation associated with senile, vascular, and Alzheimer's dementia
Inaba et al. (2011)	Cohort study	White matter lesions associated with cognitive decline and AD
Zhou et al. (2015)	Meta-analysis	Stroke increased the risk of AD
**Dementia**		
Vermeer et al. (2003)	Cohort study	The presence of silent brain infarcts more than double the risk of dementia
Newman et al. (2005)	Longitudinal cohort study	Coronary heart disease and peripheral artery disease associated with an increased risk for dementia
van Oijen et al. (2007)	Population-based, prospective cohort study	Atherosclerosis associated with an increased risk for dementia
Ikram et al. (2008)	Population-based cohort study	Men who suffered from myocardial infarction had an increased risk of dementia
Deckers et al. (2017)	Meta-analysis	Coronary heart disease associated with an increased risk for cognitive impairment and dementia
**Cognitive impairment**		
Ott et al. (1997)	Cross-sectional, population-based study	Atrial fibrillation associated with cognitive impairment and dementia
Knecht et al. (2008)	Cross-sectional	Atrial fibrillation associated with cognitive impairment and hippocampal atrophy
Roberts et al. (2010)	Population-based cohort study	Coronary heart disease associated positively with non-amnestic mild cognitive impairment
Marzona et al. (2012)	Randomized controlled trial	Atrial fibrillation associated with an increased risk of cognitive decline in the absence of overt stroke
Thong et al. (2013)	Cohort study	Silent brain infarcts associated with cognitive impairment

## Alzheimer's Disease and Atrial Fibrillation

Similarly, atrial fibrillation is another cardiovascular disease associated with an increased risk for AD. Atrial fibrillation is characterized by an irregular often rapid heart rate resulting in poor blood flow. This condition could lead to blood clots, stroke, heart failure, and other cardiovascular diseases. A diagnosis of atrial fibrillation correlated positively with cognitive impairment and dementia, with a stronger association in women, in a large cross-sectional, population-based study (Hainsworth et al., 2017). Interestingly, the association was stronger for AD with cerebrovascular disease than for vascular dementia (Ott et al., 1997). A meta-analysis of 14 studies identified a positive association between atrial fibrillation and dementia (Kwok et al., 2011). However, further analysis with patient stratification showed the association was significant in studies focusing solely on stroke (Kwok et al., 2011). Nevertheless, even in the absence of stroke, atrial fibrillation has been associated with cognitive decline and hippocampal atrophy (Knecht et al., 2008). These results were confirmed by other large studies wherein cognitive and functional decline was positively associated with atrial fibrillation in the absence of overt stroke (Bunch et al., 2010; Marzona et al., 2012).

The underlying mechanism by which atrial fibrillation is linked to AD is unknown. It has been proposed that cerebral hypoperfusion and low cardiac output resulting from atrial fibrillation cause damage to the nerve cells contributing to neurodegeneration in AD (de Bruijn and Ikram, 2014). However, it remains unknown whether atrial fibrillation contributes to neurofibrillary tangles and amyloid plaques, pathological hallmarks of AD. One study found that atrial fibrillation is associated with large ischemic lesions but not AD neuropathology (Dublin et al., 2014). The same study, however, documented that neuropathological changes associated with AD were more common in people with permanent atrial fibrillation (Dublin et al., 2014). Therefore, the evidence linking atrial fibrillation with AD neuropathology is scarce and more studies are needed to understand the underlying mechanism. The main results from epidemiological studies investigating the association between AD and atrial fibrillation are presented in Table 3.

## Alzheimer's Disease and Coronary Heart Disease

Coronary heart disease (CHD) is another condition within the cardiovascular disease spectrum that has been implicated in AD. CHD is the most common heart disease and one of the leading causes of death worldwide. There is evidence that CHD increases the risk of cognitive impairment and dementia but there are some discrepancies among the studies. For example, a longitudinal cohort study revealed that the incidence of dementia was higher in subjects with CHD, particularly in those with peripheral arterial disease, compared to normal subjects (Newman et al., 2005). This result remained significant after the exclusion of vascular dementia (Newman et al., 2005). A population-based cohort study found a positive association between CHD and non-amnestic MCI but not amnestic MCI (Roberts et al., 2010). Nevertheless, some studies have found no association between CHD and AD or dementia. A population-based case-control study including 557 dementia cases suggested that coronary artery bypass grafting was not associated with dementia or AD (Knopman et al., 2005). Similarly, a larger population-based study including 3,734 Japanese-American men failed to find a significant association between coronary artery bypass surgery and permanent cognitive impairment (Petrovitch et al., 1998). Several factors including sample size, methods, patient selection, population genetics and environmental factors may explain the differences among the studies.

The association between CHD and AD has been reinforced by larger epidemiological studies. For instance, a larger population-based cohort showed that men with unrecognized myocardial infarction had an increased risk of dementia (Ikram et al., 2008). A meta-analysis of 10 prospective cohort studies showed that CHD increased the risk of cognitive impairment and dementia (Deckers et al., 2017). Consistent with these findings, a more recent and larger meta-analysis including 16 CHD studies (1,309,483 individuals), and seven heart failure studies (1,958,702 individuals), showed a 27 and 60% increased risk of dementia among CDH and heart failure patients, respectively (Wolters et al., 2018).

Although there are some discrepancies among epidemiological studies, most of the studies suggest CHD is a risk factor for cognitive impairment and dementia. Interestingly, atherosclerosis has been suggested as the underlying mechanism linking CHD to dementia. For example, neuropathological examination in 1,000 subjects revealed that more than 77% of AD subjects had apparent circle of Willis atherosclerosis (Yarchoan et al., 2012). In addition to intracranial vessels, atherosclerosis in extracranial vessels has been linked to AD. For instance, subjects with severe carotid and femoral atherosclerosis showed a 3-fold increase risk of dementia (Hofman et al., 1997). This positive association was even stronger in subjects with both atherosclerosis and apolipoprotein epsilon 4 (APOEε4) genotype (Hofman et al., 1997). Another prospective cohort study also found a positive association between atherosclerosis and dementia but failed to identify differences among APOEε genotypes (van Oijen et al., 2007). The linkage between atherosclerosis and AD may be related to alterations in cholesterol homeostasis and inflammatory processes. Elevated serum cholesterol levels and inflammation are two main determinants in the pathogenesis of atherosclerosis and these are intimately associated with AD (Notkola et al., 1998; Casserly and Topol, 2004; Liu et al., 2020). A summary of the main findings of epidemiological studies addressing the relationship between AD and CHD is presented in Table 3.

## Alzheimer's Disease and Cardiovascular Risk Factors

In addition to cardiovascular diseases *per se*, risk factors for cardiovascular diseases including hypertension, hypercholesterolemia, and obesity have been associated with an increased risk for AD. For example, non-demented individuals with hypertension had a higher risk of severe white matter lesions compared to healthy subjects (van Dijk et al., 2004). Moreover, in a longitudinal study with 36 years of follow up, elevated systolic blood pressure in mid-life is associated with the development of neuritic plaques and neurofibrillary tangles, characteristic of AD (Petrovitch et al., 2000).

Given the link between hypertension and cognitive decline, antihypertensive drugs have been investigated as potential therapeutics for dementia. Most of the longitudinal studies have found an inverse relationship between the use of antihypertensive drugs and dementia. For example, a longitudinal study including 1,810 individuals showed that non-demented subjects taking antihypertensive drugs had a lower risk of dementia (Guo et al., 1999). Similarly, the use of any antihypertensive drug was associated with a lower incidence of AD (Khachaturian et al., 2006). Further analysis revealed that the use of potassium-sparing diuretics is associated with a greater reduction in the risk of AD (Khachaturian et al., 2006). Interestingly, another study revealed that for each year of antihypertensive treatment there was a reduction in the incidence rate of dementia compared to subjects never treated with antihypertensive drugs (Peila et al., 2006). Nonetheless, some studies showed no benefit in the use of antihypertensive drugs for cognitive decline and dementia (Morris et al., 2001; Lindsay et al., 2002; Yasar et al., 2005).

## Alzheimer's Disease and Depression

A history of depression has been associated with an increased risk of developing AD later in life. Depression is very common among the elderly and it is characterized by the loss of appetite, sleep disturbances, loss of energy, and fatigue, among many other symptoms (Ownby et al., 2006). Depression is linked to cognitive impairment and overall functional capacity in AD patients (Espiritu et al., 2001). Both depression and AD have a great impact on the quality of life and daily activities of patients. For example, a diagnosis of depression and biomarkers for AD is associated with a decline in driving performance on a road test suggesting patients with these conditions present with significant challenges when driving (Babulal et al., 2018). Earlier epidemiological studies suggested a positive association between depression and AD (Kokmen et al., 1991; Speck et al., 1995). Indeed, a case-control study claimed that depression may appear 10 years before the onset of dementia (Speck et al., 1995). These results were further supported by a later case-control study that found that the first signs of depression may appear 25 years earlier before the onset of dementia (Green et al., 2003). Collectively, these earlier studies suggested depression may be one of the earliest signs of dementia. Nevertheless, other case-control studies did not find a significant association between both diseases (French et al., 1985; Broe et al., 1990).

Likewise, cohort studies identified a significant association between depression and a greater risk of dementia. A retrospective cohort study including 19,000 patients supported the hypothesis that depression is a predictor of future dementia (Buntinx et al., 1996). Similarly, another study showed a positive association between depressed mood and risk for dementia (Devanand et al., 1996). Furthermore, a systematic review and meta-analysis of both case controls and cohort studies identified a positive association between depression and increased risk for AD (Ownby et al., 2006). Of note, this study indicated that depression, rather than a prodromal symptom, may be a risk factor for AD. The main results from epidemiological studies investigating the association between AD and depression are presented in Table 4.

**Table 4:** Studies investigating the association between depression, dementia, and AD.

**Study**	**Study design**	**Main results**
**Alzheimer's disease**		
Kokmen et al. (1991)	Population-based case-control study	Episodic depression associated positively with AD
Devanand et al. (1996)	Prospective longitudinal study	Depressed mood moderately increased the risk for AD
Green et al. (2003)	Cross-sectional, case-control study	Depression symptoms may occur 25 years before the onset of AD
Ownby et al. (2006)	Systematic review, meta-analysis	Depression associated with an increased risk of AD
**Dementia**		
Speck et al. (1995)	Case-control study	Depression may occur 10 years before the onset of dementia
Buntinx et al. (1996)	Retrospective cohort study	Old age depression may be a predictor of subsequent dementia

The mechanisms underlying the association between depression and AD are not well-understood. Very few studies have identified shared genetic risk factors between both diseases including APOEε (Stewart et al., 2001) and complement receptor 1 (CR1) (Hamilton et al., 2012), however, there are some discrepancies (Zubenko et al., 1996; Mauricio et al., 2000). Recently, a larger genome-wide association study did not identify shared genetic variants between depression and AD (Gibson et al., 2017). These findings suggest that the underlying mechanism explaining the association between depression and AD may not be explained by shared genetic factors.

Another potential mechanism linking both depression and AD may be related to inflammation and vascular disease. In this regard, higher levels of TNF and apoptotic signaling ligand FAS have been documented in patients with depression and heart disease (Parissis et al., 2004). Moreover, the upregulation of proinflammatory cytokines associated with depression, atherosclerosis, and subsequent coronary heart disease in women (Suarez et al., 2004). The connection between depression and inflammation has been further reinforced by the finding that treatment with antidepressants resulted in the alteration of pro and anti-inflammatory cytokines (Castanon et al., 2002). Conversely, treatment with anti-inflammatory drugs and cytokine inhibitors have elicited antidepressant effects (Kohler et al., 2016). Collectively, these results suggest that inflammatory processes may be intimately related to the development of depression and the use of anti-inflammatories may be a potential therapeutic strategy against depression.

## Alzheimer's Disease and the Gut Microbiome

The human gastrointestinal tract is home to trillions of microorganisms collectively called the gut microbiome. Dysbiosis of the human gut microbiome has been linked to numerous diseases including respiratory, metabolic, autoimmune, and neurodegenerative diseases (Lynch and Pedersen, 2016; Dinan and Cryan, 2017). This is not surprising since the gut microbiome influences not only nutrient metabolism but it is also intimately related to the immune system and brain development. Normal flora contributes to the production of neuroactive molecules including serotonin, GABA, acetylcholine, histamine, tryptophan, and catecholamines (Dinan and Cryan, 2017). For example, alterations in tryptophan metabolism through the kynurenine pathway have been linked to AD (Giil et al., 2017). Because the gut microbiome is known to play a role in autoimmunity, neuroinflammation, and neurogenesis in the brain, a gut-brain axis of neurodegeneration has been implicated in the pathogenesis of AD and other neurodegenerative diseases (Fung et al., 2017). Biochemical studies showed that *Escherichia coli* can produce amyloid fibers and regulate amyloidosis (Chapman et al., 2002). Also, disturbances to the microbiome homeostasis by drugs and diet may increase pathogen susceptibility and inflammation. For example, a systematic review suggested that antibiotic use was associated with severe dementia (van der Maaden et al., 2015). Interestingly, a randomized double-blind and controlled clinical trial showed the efficacy of probiotic treatment in improving cognitive function in AD patients (Akbari et al., 2016). Nevertheless, because of the small sample size used in this trial, further studies are needed to verify these findings.

Accumulating evidence from epidemiological studies suggests that inflammatory bowel disease (IBD) is associated with an increased risk of dementia. A population-based study including 32,298 patients with irritable bowel syndrome showed an increased risk of dementia in patients older than 50 years (Chen et al., 2016). Furthermore, a cohort study of 1,742 patients with IBD showed a significant positive association between IBD and subsequent development of dementia (Zhang et al., 2020). These findings are supported by a recent meta-analysis that found a positive association between IBD and subsequent development of AD (Fu et al., 2020).

Crohn's disease is another gastrointestinal disease that has been implicated in AD. One study identified a common genetic factor between Crohn's' disease and AD. A genetic variant near the IPMK gene, associated with Crohn's disease (O'Donnell et al., 2019), was found to increase the risk of AD (Yokoyama et al., 2016). The genetic overlap between these diseases and AD is not substantial and thus unlikely to explain the comorbidity between AD and inflammatory gut diseases. The exact mechanisms by which gastrointestinal diseases are linked to AD are unknown, but the consensus among the studies suggest that disruption in the gut microbiome can lead to the production of toxic metabolites that can infiltrate through the blood-brain barrier and cause widespread neuroinflammation. The main results from the studies investigating the association between AD and gastrointestinal diseases are presented in Table 5.

**Table 5:** Studies investigating the association between the gut microbiome, dementia and AD.

**Study**	**Study design**	**Main results**
**Alzheimer's disease**		
Akbari et al. (2016)	Randomized double-blind controlled trial	Probiotic treatment improved cognitive function in AD patients
Yokoyama et al. (2016)	Genome-wide association study	A genetic variant near IMPK is shared between Crohn's disease and AD
Giil et al. (2017)	Case-control	Plasma levels of several kynurenines were lower in AD compared to controls
Fu et al. (2020)	Systematic review, meta-analysis	Inflammatory bowel disease associated with an increased risk for AD
**Dementia**		
van der Maaden et al. (2015)	Systematic review	The use of antibiotics may be associated with dementia
Chen et al. (2016)	Population-based study	Inflammatory bowel disease associated with an increased risk for dementia
Zhang et al. (2020)	Longitudinal cohort study	Inflammatory bowel disease associated with an increased risk for dementia

## Bioinformatic Approaches to Understanding Comorbidities in AD

Bioinformatic-based studies have laid the groundwork for the discovery of dysregulated biological pathways, therapeutic targets, and biomarkers, in neurodegenerative diseases (Santiago et al., 2017). Network biology approaches have been useful in identifying shared and unique pathways between AD and other diseases. In the context of diabetes, network analysis of transcriptomic data from AD and T2D brains revealed a central role for autophagy in the molecular linking of both diseases (Caberlotto et al., 2019). Another study using blood transcriptomic data showed that shared networks between MCI and T2D were related to inflammation whereas those networks shared between advanced AD and T2D were associated with the impairment in insulin signaling and defective cardiovascular system (Santiago et al., 2019).

In regards to cardiovascular and gut-related diseases, some bioinformatic studies have explored the connection with dementia. For example, one study showed that genes associated with AD such as apolipoprotein E (*APOE)*, alpha 2 macroglobulin (*A2M)*, paraoxonase 2 (*PON2)*, and microtubule-associated protein 4 (*MAP4)*, were closely related to genes associated with cardiovascular disease, including catechol-O-methyltransferase (*COMT)*, cystathionine beta synthase (*CBS)*, and WNK lysine deficient protein kinase 1 (WNK1) suggesting both diseases are linked through shared molecular networks (Ray et al., 2008). Weighted gene coexpression network analysis of proteomic data from over 400 postmortem brains with AD identified 23 shared proteins between AD and cerebral atherosclerosis and suggested that cerebral atherosclerosis contributed to dementia risk through decreased synaptic signaling and regulation and increased myelination (https://doi.org/10.1101/793349). In the context of gastrointestinal diseases, an integrative meta-analysis of 3 microarrays from patients with Crohn's disease identified ELAV-like RNA binding protein 1 (ELAVL1) and APP as the most significantly, upregulated and downregulated, respectively, in the blood of patients with Crohn's disease (Li et al., 2020). Interestingly, both genes have been linked to AD. For instance, APP is central to the pathogenesis of AD (O'Brien and Wong, 2011) and mutations in *APP* are known to cause familial AD (Weggen and Beher, 2012). Understanding APP metabolism and processing has been key to better understand the pathogenesis of AD (O'Brien and Wong, 2011). *ELAVL1* has been reported in AD, and its alteration may be related to APP processing (Amadio et al., 2009). To the best of our knowledge, bioinformatic-based studies exploring the association between inflammatory bowel disease and AD are not currently available. Future studies investigating the shared molecular networks between inflammatory bowel disease and AD will be important for identifying potential mechanisms and therapeutic targets.

## Conclusions

Several comorbidities associated with AD may be involved in the disease pathogenesis and progression and thus, may have important clinical implications in the management of patients. For example, disease comorbidities like T2D and depression are associated with poor prognosis in AD patients (Li J. Q. et al., 2016). Therefore, it is important to carefully address the presence of comorbid diseases in AD in order to provide personalized treatment.

Despite the substantial evidence provided by epidemiological studies regarding the linkage between AD and some of its comorbidities, the precise mechanism explaining their coexistence with AD is still poorly understood. Epidemiological studies are useful in providing the basis for understanding disease risk factors and comorbidities but causal relationships are more difficult to disentangle. Because of the shared genetics, environmental factors, and strong competing risk of death, the mechanisms underlying the association between AD, dementia, and other diseases remain a challenging task for scientists and clinicians. Integrative bioinformatic approaches combining epidemiological, genetic, transcriptomic, proteomic, and metabolomic data may be key to better understand comorbidities in AD (Santiago and Potashkin, 2014).

Inflammation appears to be a central mechanism linking AD with other chronic diseases, however, it remains unclear whether inflammation plays a causative role or it is a consequence of neurodegeneration (Pugazhenthi et al., 2017). Most of the research indicates a bidirectional association between inflammation and AD (Newcombe et al., 2018). Several studies have suggested that neuroinflammation is fundamental in the pathogenesis of AD and contributes as much as do Aβ plaques and NFT (Heneka et al., 2015). Furthermore, the presence of AD pathological features in cognitively normal individuals suggest that multiple factors are required for the progression to AD (29876101). Neuroinflammation has been proposed as one of the earliest events preceding AD (20160456). For example, elevated levels of inflammatory cytokines IL-18, TNFα, and IFNγ have been shown to increase Aβ production in AD cellular models (Blasko et al., 1999; Sutinen et al., 2012). These inflammatory cytokines activate microglia, the resident phagocytes of the brain, which aid in the clearance of Aβ (Paresce et al., 1996; Bamberger et al., 2003). In this context, persistent neuroinflammation results in a decrease in the microglia phagocytic capacity leading to the accumulation of toxic Aβ (Krabbe et al., 2013). These studies reinforce the hypothesis that neuroinflammation may be an initial trigger in the neurodegenerative cascade in AD.

While some genetic factors are strongly connected with the development of late-onset AD, genetics alone does not explain the vast majority of the AD cases. The fact that many of the comorbidities associated with AD are related to dysregulated metabolic pathways implies that lifestyle factors play a role in the disease pathogenesis. In this regard, lifestyle modifications including exercise and diet may interact with genetic susceptibility genes and improve cognitive abilities in AD patients (Liang et al., 2018; Jensen et al., 2019). For example, physical exercise elicited a greater positive effect in cognitive function in AD patients who were APOEε carriers compared to non-carriers (Jensen et al., 2019). Moreover, APOEε carriers with a sedentary lifestyle showed a greater amyloid beta deposition compared to non-carriers (Head et al., 2012).

In the context of diet, adherence to a Mediterranean diet has been associated with a decreased risk for AD (van den Brink et al., 2019). Similar to physical exercise, genetic-diet interactions have been documented in AD patients. For example, a Mediterranean diet showed beneficial effects in cognitive function in AD patients that were carriers of several genetic variants in genes including *CRI, CLU*, and *PICALM* but not in APOEε carriers (Martinez-Lapiscina et al., 2014). Also, a ketogenic diet has been investigated as a potential therapeutic strategy in AD patients. Several clinical trials on ketogenic diets have shown promising results in improving cognitive function in AD patients (Henderson et al., 2009; Taylor et al., 2018). Interestingly, APOEε carriers were less responsive to a ketogenic diet compared to non-carriers (Reger et al., 2004). Collectively, these results illustrate the close interaction between genetic and environmental factors in modifying an individual disease risk for AD. While some lifestyle modifications such as exercise and diet, may be beneficial for AD patients, other factors including comorbidities and genetic profiles should be taken into consideration when evaluating treatments.

## Author Contributions

JS and JP wrote and edited the manuscript. All authors contributed to the article and approved the submitted version.

## Conflict of Interest

JS is employed by the company NeuroHub Analytics, LLC. The remaining author declares that the research was conducted in the absence of any commercial or financial relationships that could be construed as a potential conflict of interest.

## References

[B1] Akbari E. Asemi Z. Daneshvar Kakhaki R. Bahmani F. Kouchaki E. Tamtaji O. R.. (2016). Effect of probiotic supplementation on cognitive function and metabolic status in Alzheimer's disease: a randomized, double-blind and controlled trial. Front. Aging Neurosci. 8:256. 10.3389/fnagi.2016.00256 27891089 PMC5105117

[B2] Akomolafe A. Beiser A. Meigs J. B. Au R. Green R. C. Farrer L. A.. (2006). Diabetes mellitus and risk of developing Alzheimer disease: results from the Framingham Study. Arch. Neurol. 63, 1551–1555. 10.1001/archneur.63.11.1551 17101823

[B3] Albert M. S. DeKosky S. T. Dickson D. Dubois B. Feldman H. H. Fox N. C.. (2011). The diagnosis of mild cognitive impairment due to Alzheimer's disease: recommendations from the National Institute on Aging-Alzheimer's Association workgroups on diagnostic guidelines for Alzheimer's disease. Alzheimers. Dement. 7, 270–279. 10.1016/j.jalz.2011.03.008 21514249 PMC3312027

[B4] Alzheimer's Association. (2016). 2016 Alzheimer's disease facts and figures. Alzheimers. Dement. 12, 459–509. 10.1016/j.jalz.2016.03.001 27570871

[B5] Amadio M. Pascale A. Wang J. Ho L. Quattrone A. Gandy S.. (2009). nELAV proteins alteration in Alzheimer's disease brain: a novel putative target for amyloid-beta reverberating on AbetaPP processing. J. Alzheimers. Dis. 16, 409–419. 10.3233/JAD-2009-0967 19221430 PMC6057145

[B6] Athauda D. Maclagan K. Skene S. S. Bajwa-Joseph M. Letchford D. Chowdhury K.. (2017). Exenatide once weekly versus placebo in Parkinson's disease: a randomised, double-blind, placebo-controlled trial. Lancet 390, 1664–1675. 10.1016/S0140-6736(17)31585-4 28781108 PMC5831666

[B7] Aviles-Olmos I. Dickson J. Kefalopoulou Z. Djamshidian A. Ell P. Soderlund T.. (2013). Exenatide and the treatment of patients with Parkinson's disease. J. Clin. Invest. 123, 2730–2736. 10.1172/JCI68295 23728174 PMC3668846

[B8] Babulal G. M. Chen S. Williams M. M. Trani J. F. Bakhshi P. Chao G. L.. (2018). Depression and Alzheimer's disease biomarkers predict driving decline. J. Alzheimers. Dis. 66, 1213–1221. 10.3233/JAD-180564 30400098 PMC6330210

[B9] Bamberger M. E. Harris M. E. McDonald D. R. Husemann J. Landreth G. E. (2003). A cell surface receptor complex for fibrillar beta-amyloid mediates microglial activation. J. Neurosci. 23, 2665–2674. 10.1523/JNEUROSCI.23-07-02665.2003 12684452 PMC6742111

[B10] Biessels G. J. Staekenborg S. Brunner E. Brayne C. Scheltens P. (2006). Risk of dementia in diabetes mellitus: a systematic review. Lancet Neurol. 5, 64–74. 10.1016/S1474-4422(05)70284-2 16361024

[B11] Blasko I. Marx F. Steiner E. Hartmann T. Grubeck-Loebenstein B. (1999). TNFalpha plus IFNgamma induce the production of Alzheimer beta-amyloid peptides and decrease the secretion of APPs. FASEB J. 13, 63–68. 10.1096/fasebj.13.1.63 9872930

[B12] Bloom G. S. (2014). Amyloid-beta and tau: the trigger and bullet in Alzheimer disease pathogenesis. JAMA Neurol. 71, 505–508. 10.1001/jamaneurol.2013.5847 24493463 PMC12908160

[B13] Boni-Schnetzler M. Thorne J. Parnaud G. Marselli L. Ehses J. A. Kerr-Conte J.. (2008). Increased interleukin (IL)-1beta messenger ribonucleic acid expression in beta -cells of individuals with type 2 diabetes and regulation of IL-1beta in human islets by glucose and autostimulation. J. Clin. Endocrinol. Metab. 93, 4065–4074. 10.1210/jc.2008-0396 18664535 PMC2579638

[B14] Broe G. A. Henderson A. S. Creasey H. McCusker E. Korten A. E. Jorm A. F.. (1990). A case-control study of Alzheimer's disease in Australia. Neurology 40, 1698–1707. 10.1212/WNL.40.11.1698 2146525

[B15] Bunch T. J. Weiss J. P. Crandall B. G. May H. T. Bair T. L. Osborn J. S.. (2010). Atrial fibrillation is independently associated with senile, vascular, and Alzheimer's dementia. Heart Rhythm. 7, 433–437. 10.1016/j.hrthm.2009.12.004 20122875

[B16] Buntinx F. Kester A. Bergers J. Knottnerus J. A. (1996). Is depression in elderly people followed by dementia? A retrospective cohort study based in general practice. Age Ageing 25, 231–233. 10.1093/ageing/25.3.231 8670559

[B17] Caberlotto L. Nguyen T. P. Lauria M. Priami C. Rimondini R. Maioli S.. (2019). Cross-disease analysis of Alzheimer's disease and type-2 Diabetes highlights the role of autophagy in the pathophysiology of two highly comorbid diseases. Sci. Rep. 9:3965. 10.1038/s41598-019-39828-5 30850634 PMC6408545

[B18] Campbell J. M. Stephenson M. D. de Courten B. Chapman I. Bellman S. M. Aromataris E. (2018). Metformin use associated with reduced risk of dementia in patients with diabetes: a systematic review and meta-analysis. J. Alzheimers. Dis. 65, 1225–1236. 10.3233/JAD-180263 30149446 PMC6218120

[B19] Casserly I. Topol E. (2004). Convergence of atherosclerosis and Alzheimer's disease: inflammation, cholesterol, and misfolded proteins. Lancet 363, 1139–1146. 10.1016/S0140-6736(04)15900-X 15064035

[B20] Castanon N. Leonard B. E. Neveu P. J. Yirmiya R. (2002). Effects of antidepressants on cytokine production and actions. Brain Behav. Immun. 16, 569–574. 10.1016/S0889-1591(02)00008-9 12401470

[B21] Chapman M. R. Robinson L. S. Pinkner J. S. Roth R. Heuser J. Hammar M.. (2002). Role of *Escherichia coli* curli operons in directing amyloid fiber formation. Science 295, 851–855. 10.1126/science.1067484 11823641 PMC2838482

[B22] Chatterjee S. Mudher A. (2018). Alzheimer's disease and Type 2 diabetes: a critical assessment of the shared pathological traits. Front. Neurosci. 12:383. 10.3389/fnins.2018.00383 29950970 PMC6008657

[B23] Chen C. H. Lin C. L. Kao C. H. (2016). Irritable bowel syndrome is associated with an increased risk of dementia: a nationwide population-based study. PLoS ONE 11:e0144589. 10.1371/journal.pone.0144589 26731277 PMC4701489

[B24] Cheng G. Huang C. Deng H. Wang H. (2012). Diabetes as a risk factor for dementia and mild cognitive impairment: a meta-analysis of longitudinal studies. Intern. Med. J. 42, 484–491. 10.1111/j.1445-5994.2012.02758.x 22372522

[B25] Cooper G. J. Willis A. C. Clark A. Turner R. C. Sim R. B. Reid K. B. (1987). Purification and characterization of a peptide from amyloid-rich pancreases of type 2 diabetic patients. Proc. Natl. Acad. Sci. U.S.A. 84, 8628–8632. 10.1073/pnas.84.23.8628 3317417 PMC299599

[B26] de Bruijn R. F. Ikram M. A. (2014). Cardiovascular risk factors and future risk of Alzheimer's disease. BMC Med. 12:130. 10.1186/s12916-014-0130-5 25385322 PMC4226863

[B27] de la Monte S. M. (2019). The full spectrum of Alzheimer's disease is rooted in metabolic derangements that drive Type 3 diabetes. Adv. Exp. Med. Biol. 1128, 45–83. 10.1007/978-981-13-3540-2_4 31062325 PMC9996398

[B28] Deckers K. Schievink S. H. J. Rodriquez M. M. F. van Oostenbrugge R. J. van Boxtel M. P. J. Verhey F. R. J.. (2017). Coronary heart disease and risk for cognitive impairment or dementia: systematic review and meta-analysis. PLoS ONE 12:e0184244. 10.1371/journal.pone.0184244 28886155 PMC5590905

[B29] Decourt B. Lahiri D. K. Sabbagh M. N. (2017). Targeting tumor necrosis factor alpha for Alzheimer's disease. Curr. Alzheimer Res. 14, 412–425. 10.2174/1567205013666160930110551 27697064 PMC5328927

[B30] Devanand D. P. Sano M. Tang M. X. Taylor S. Gurland B. J. Wilder D.. (1996). Depressed mood and the incidence of Alzheimer's disease in the elderly living in the community. Arch. Gen. Psychiatry 53, 175–182. 10.1001/archpsyc.1996.01830020093011 8629893

[B31] Dinan T. G. Cryan J. F. (2017). The microbiome-gut-brain axis in health and disease. Gastroenterol. Clin. North Am. 46, 77–89. 10.1016/j.gtc.2016.09.007 28164854

[B32] Dublin S. Anderson M. L. Heckbert S. R. Hubbard R. A. Sonnen J. A. Crane P. K.. (2014). Neuropathologic changes associated with atrial fibrillation in a population-based autopsy cohort. J. Gerontol. A Biol. Sci. Med. Sci. 69, 609–615. 10.1093/gerona/glt141 24077599 PMC3991149

[B33] Espiritu D. A. Rashid H. Mast B. T. Fitzgerald J. Steinberg J. Lichtenberg P. A. (2001). Depression, cognitive impairment and function in Alzheimer's disease. Int. J. Geriatr. Psychiatry 16, 1098–1103. 10.1002/gps.476 11746657

[B34] Fontbonne A. Berr C. Ducimetiere P. Alperovitch A. (2001). Changes in cognitive abilities over a 4-year period are unfavorably affected in elderly diabetic subjects: results of the Epidemiology of Vascular Aging Study. Diabetes Care 24, 366–370. 10.2337/diacare.24.2.366 11213894

[B35] French L. R. Schuman L. M. Mortimer J. A. Hutton J. T. Boatman R. A. Christians B. (1985). A case-control study of dementia of the Alzheimer type. Am. J. Epidemiol. 121, 414–421. 10.1093/oxfordjournals.aje.a114013 4014131

[B36] Fu P. Gao M. Yung K. K. L. (2020). Association of intestinal disorders with Parkinson's disease and Alzheimer's Disease: a systematic review and meta-analysis. ACS Chem. Neurosci. 11, 395–405. 10.1021/acschemneuro.9b00607 31876406

[B37] Fung T. C. Olson C. A. Hsiao E. Y. (2017). Interactions between the microbiota, immune and nervous systems in health and disease. Nat. Neurosci. 20, 145–155. 10.1038/nn.4476 28092661 PMC6960010

[B38] Gabbouj S. Ryhanen S. Marttinen M. Wittrahm R. Takalo M. Kemppainen S.. (2019). altered insulin signaling in Alzheimer's disease brain - special emphasis on PI3K-Akt pathway. Front. Neurosci. 13:629. 10.3389/fnins.2019.00629 31275108 PMC6591470

[B39] Geijselaers S. L. C. Sep S. J. S. Claessens D. Schram M. T. van Boxtel M. P. J. Henry R. M. A.. (2017). The Role of hyperglycemia, insulin resistance, and blood pressure in diabetes-associated differences in cognitive performance-the maastricht study. Diabetes Care 40, 1537–1547. 10.2337/dc17-0330 28842522

[B40] Gejl M. Brock B. Egefjord L. Vang K. Rungby J. Gjedde A. (2017). Blood-brain glucose transfer in Alzheimer's disease: effect of GLP-1 analog treatment. Sci. Rep. 7:17490. 10.1038/s41598-017-17718-y 29235507 PMC5727512

[B41] Gibson J. Russ T. C. Adams M. J. Clarke T. K. Howard D. M. Hall L. S.. (2017). Assessing the presence of shared genetic architecture between Alzheimer's disease and major depressive disorder using genome-wide association data. Transl. Psychiatry 7:e1094. 10.1038/tp.2017.49 28418403 PMC5416691

[B42] Giil L. M. Midttun O. Refsum H. Ulvik A. Advani R. Smith A. D.. (2017). Kynurenine pathway metabolites in Alzheimer's disease. J. Alzheimers. Dis. 60, 495–504. 10.3233/JAD-170485 28869479

[B43] Green R. C. Cupples L. A. Kurz A. Auerbach S. Go R. Sadovnick D.. (2003). Depression as a risk factor for Alzheimer disease: the MIRAGE study. Arch. Neurol. 60, 753–759. 10.1001/archneur.60.5.753 12756140

[B44] Guo X. Geng M. Du G. (2005). Glucose transporter 1, distribution in the brain and in neural disorders: its relationship with transport of neuroactive drugs through the blood-brain barrier. Biochem. Genet. 43, 175–187. 10.1007/s10528-005-1510-5 15932065

[B45] Guo Z. Fratiglioni L. Zhu L. Fastbom J. Winblad B. Viitanen M. (1999). Occurrence and progression of dementia in a community population aged 75 years and older: relationship of antihypertensive medication use. Arch. Neurol. 56, 991–996. 10.1001/archneur.56.8.991 10448805

[B46] Hainsworth A. H. Minett T. Andoh J. Forster G. Bhide I. Barrick T. R.. (2017). Neuropathology of white matter lesions, blood-brain barrier dysfunction, and dementia. Stroke 48, 2799–2804. 10.1161/STROKEAHA.117.018101 28855392 PMC5986073

[B47] Hamilton G. Evans K. L. Macintyre D. J. Deary I. J. Dominiczak A. Smith B. H.. (2012). Alzheimer's disease risk factor complement receptor 1 is associated with depression. Neurosci. Lett. 510, 6–9. 10.1016/j.neulet.2011.12.059 22244847

[B48] Hassing L. B. Johansson B. Nilsson S. E. Berg S. Pedersen N. L. Gatz M.. (2002). Diabetes mellitus is a risk factor for vascular dementia, but not for Alzheimer's disease: a population-based study of the oldest old. Int. Psychogeriatr. 14, 239–248. 10.1017/S104161020200844X 12475085

[B49] Head D. Bugg J. M. Goate A. M. Fagan A. M. Mintun M. A. Benzinger T.. (2012). Exercise engagement as a moderator of the effects of APOE genotype on amyloid deposition. Arch. Neurol. 69, 636–643. 10.1001/archneurol.2011.845 22232206 PMC3583203

[B50] Henderson S. T. Vogel J. L. Barr L. J. Garvin F. Jones J. J. Costantini L. C. (2009). Study of the ketogenic agent AC-1202 in mild to moderate Alzheimer's disease: a randomized, double-blind, placebo-controlled, multicenter trial. Nutr. Metab. 6:31. 10.1186/1743-7075-6-31 19664276 PMC2731764

[B51] Heneka M. T. Carson M. J. El Khoury J. Landreth G. E. Brosseron F. Feinstein D. L.. (2015). Neuroinflammation in Alzheimer's disease. Lancet Neurol. 14, 388–405. 10.1016/S1474-4422(15)70016-5 25792098 PMC5909703

[B52] Hofman A. Ott A. Breteler M. M. Bots M. L. Slooter A. J. van Harskamp F.. (1997). Atherosclerosis, apolipoprotein E, and prevalence of dementia and Alzheimer's disease in the Rotterdam Study. Lancet 349, 151–154. 10.1016/S0140-6736(96)09328-2 9111537

[B53] Holscher C. (2018). Novel dual GLP-1/GIP receptor agonists show neuroprotective effects in Alzheimer's and Parkinson's disease models. Neuropharmacology 136, 251–259. 10.1016/j.neuropharm.2018.01.040 29402504

[B54] Hotamisligil G. S. Shargill N. S. Spiegelman B. M. (1993). Adipose expression of tumor necrosis factor-alpha: direct role in obesity-linked insulin resistance. Science 259, 87–91. 10.1126/science.7678183 7678183

[B55] Ikram M. A. van Oijen M. de Jong F. J. Kors J. A. Koudstaal P. J. Hofman A.. (2008). Unrecognized myocardial infarction in relation to risk of dementia and cerebral small vessel disease. Stroke 39, 1421–1426. 10.1161/STROKEAHA.107.501106 18323497

[B56] Imfeld P. Bodmer M. Jick S. S. Meier C. R. (2012). Metformin, other antidiabetic drugs, and risk of Alzheimer's disease: a population-based case-control study. J. Am. Geriatr. Soc. 60, 916–921. 10.1111/j.1532-5415.2012.03916.x 22458300

[B57] Inaba M. White L. Bell C. Chen R. Petrovitch H. Launer L.. (2011). White matter lesions on brain magnetic resonance imaging scan and 5-year cognitive decline: the Honolulu-Asia aging study. J. Am. Geriatr. Soc. 59, 1484–1489. 10.1111/j.1532-5415.2011.03490.x 21718274 PMC5201137

[B58] Italiani P. Puxeddu I. Napoletano S. Scala E. Melillo D. Manocchio S.. (2018). Circulating levels of IL-1 family cytokines and receptors in Alzheimer's disease: new markers of disease progression? J. Neuroinflammation. 15, 342. 10.1186/s12974-018-1376-1 30541566 PMC6292179

[B59] Janson J. Laedtke T. Parisi J. E. O'Brien P. Petersen R. C. Butler P. C. (2004). Increased risk of type 2 diabetes in Alzheimer disease. Diabetes 53, 474–481. 10.2337/diabetes.53.2.474 14747300

[B60] Jensen C. S. Simonsen A. H. Siersma V. Beyer N. Frederiksen K. S. Gottrup H.. (2019). Patients with Alzheimer's disease who carry the APOE epsilon4 allele benefit more from physical exercise. Alzheimers Dement. 5, 99–106. 10.1016/j.trci.2019.02.007 31011620 PMC6461575

[B61] Kandimalla R. Thirumala V. Reddy P. H. (2017). Is Alzheimer's disease a Type 3 diabetes? A critical appraisal. Biochim Biophys Acta Mol Basis Dis. 1863, 1078–1089. 10.1016/j.bbadis.2016.08.018 27567931 PMC5344773

[B62] Khachaturian A. S. Zandi P. P. Lyketsos C. G. Hayden K. M. Skoog I. Norton M. C.. (2006). Antihypertensive medication use and incident Alzheimer disease: the Cache County Study. Arch. Neurol. 63, 686–692. 10.1001/archneur.63.5.noc60013 16533956

[B63] Knecht S. Oelschlager C. Duning T. Lohmann H. Albers J. Stehling C.. (2008). Atrial fibrillation in stroke-free patients is associated with memory impairment and hippocampal atrophy. Eur. Heart J. 29, 2125–2132. 10.1093/eurheartj/ehn341 18667399

[B64] Knopman D. S. Petersen R. C. Cha R. H. Edland S. D. Rocca W. A. (2005). Coronary artery bypass grafting is not a risk factor for dementia or Alzheimer disease. Neurology 65, 986–990. 10.1212/01.WNL.0000171954.92119.c7 16217048

[B65] Koenig A. M. Mechanic-Hamilton D. Xie S. X. Combs M. F. Cappola A. R. Xie L.. (2017). Effects of the insulin sensitizer metformin in Alzheimer disease: pilot data from a randomized placebo-controlled crossover study. Alzheimer Dis. Assoc. Disord. 31, 107–113. 10.1097/WAD.0000000000000202 28538088 PMC5476214

[B66] Kohler O. Krogh J. Mors O. Benros M. E. (2016). Inflammation in depression and the potential for anti-inflammatory treatment. Curr. Neuropharmacol. 14, 732–742. 10.2174/1570159X14666151208113700 27640518 PMC5050394

[B67] Kokmen E. Beard C. M. Chandra V. Offord K. P. Schoenberg B. S. Ballard D. J. (1991). Clinical risk factors for Alzheimer's disease: a population-based case-control study. Neurology 41, 1393–1397. 10.1212/WNL.41.9.1393 1891088

[B68] Krabbe G. Halle A. Matyash V. Rinnenthal J. L. Eom G. D. Bernhardt U.. (2013). Functional impairment of microglia coincides with Beta-amyloid deposition in mice with Alzheimer-like pathology. PLoS ONE 8:e60921. 10.1371/journal.pone.0060921 23577177 PMC3620049

[B69] Kuan Y. C. Huang K. W. Lin C. L. Hu C. J. Kao C. H. (2017). Effects of metformin exposure on neurodegenerative diseases in elderly patients with type 2 diabetes mellitus. Prog. Neuropsychopharmacol. Biol. Psychiatry 79, 77–83. 10.1016/j.pnpbp.2017.06.002 28583443

[B70] Kwok C. S. Loke Y. K. Hale R. Potter J. F. Myint P. K. (2011). Atrial fibrillation and incidence of dementia: a systematic review and meta-analysis. Neurology 76, 914–922. 10.1212/WNL.0b013e31820f2e38 21383328

[B71] Lai K. S. P. Liu C. S. Rau A. Lanctot K. L. Kohler C. A. Pakosh M.. (2017). Peripheral inflammatory markers in Alzheimer's disease: a systematic review and meta-analysis of 175 studies. J. Neurol. Neurosurg. Psychiatr. 88, 876–882. 10.1136/jnnp-2017-316201 28794151

[B72] Li H. Li Q. Sun S. Lei P. Cai X. Shen G. (2020). Integrated bioinformatics analysis identifies ELAVL1 and APP as candidate crucial genes for Crohn's disease. J Immunol. Res. 2020:3067273. 10.1155/2020/3067273 32724827 PMC7382743

[B73] Li J. Q. Tan L. Wang H. F. Tan M. S. Tan L. Xu W.. (2016). Risk factors for predicting progression from mild cognitive impairment to Alzheimer's disease: a systematic review and meta-analysis of cohort studies. J. Neurol. Neurosurg. Psychiatr. 87, 476–484. 10.1136/jnnp-2014-310095 26001840

[B74] Li W. Risacher S. L. Huang E. Saykin A. J. Alzheimer's Disease Neuroimaging I. (2016). Type 2 diabetes mellitus is associated with brain atrophy and hypometabolism in the ADNI cohort. Neurology 87, 595–600. 10.1212/WNL.0000000000002950 27385744 PMC4977372

[B75] Liang J. H. Xu Y. Lin L. Jia R. X. Zhang H. B. Hang L. (2018). Comparison of multiple interventions for older adults with Alzheimer disease or mild cognitive impairment: a PRISMA-compliant network meta-analysis. Medicine 97:e10744. 10.1097/MD.0000000000010744 29768349 PMC5976284

[B76] Liao Y. F. Wang B. J. Cheng H. T. Kuo L. H. Wolfe M. S. (2004). Tumor necrosis factor-alpha, interleukin-1beta, and interferon-gamma stimulate gamma-secretase-mediated cleavage of amyloid precursor protein through a JNK-dependent MAPK pathway. J. Biol. Chem. 279, 49523–49532. 10.1074/jbc.M402034200 15347683

[B77] Lindsay J. Laurin D. Verreault R. Hebert R. Helliwell B. Hill G. B.. (2002). Risk factors for Alzheimer's disease: a prospective analysis from the Canadian Study of Health and Aging. Am. J. Epidemiol. 156, 445–453. 10.1093/aje/kwf074 12196314

[B78] Liu Y. Liu F. Grundke-Iqbal I. Iqbal K. Gong C. X. (2009). Brain glucose transporters, O-GlcNAcylation and phosphorylation of tau in diabetes and Alzheimer's disease. J. Neurochem. 111, 242–249. 10.1111/j.1471-4159.2009.06320.x 19659459 PMC2760012

[B79] Liu Y. Liu F. Grundke-Iqbal I. Iqbal K. Gong C. X. (2011). Deficient brain insulin signalling pathway in Alzheimer's disease and diabetes. J. Pathol. 225, 54–62. 10.1002/path.2912 21598254 PMC4484598

[B80] Liu Y. Liu F. Iqbal K. Grundke-Iqbal I. Gong C. X. (2008). Decreased glucose transporters correlate to abnormal hyperphosphorylation of tau in Alzheimer disease. FEBS Lett. 582, 359–364. 10.1016/j.febslet.2007.12.035 18174027 PMC2247364

[B81] Liu Y. Zhong X. Shen J. Jiao L. Tong J. Zhao W.. (2020). Elevated serum TC and LDL-C levels in Alzheimer's disease and mild cognitive impairment: a meta-analysis study. Brain Res. 1727:146554. 10.1016/j.brainres.2019.146554 31765631

[B82] Lynch S. V. Pedersen O. (2016). The Human Intestinal Microbiome in Health and Disease. N. Engl. J. Med. 375, 2369–2379. 10.1056/NEJMra1600266 27974040

[B83] Macauley S. L. Stanley M. Caesar E. E. Yamada S. A. Raichle M. E. Perez R.. (2015). Hyperglycemia modulates extracellular amyloid-beta concentrations and neuronal activity *in vivo*. J. Clin. Invest. 125, 2463–2467. 10.1172/JCI79742 25938784 PMC4497756

[B84] MacKnight C. Rockwood K. Awalt E. McDowell I. (2002). Diabetes mellitus and the risk of dementia, Alzheimer's disease and vascular cognitive impairment in the Canadian Study of Health and Aging. Dement. Geriatr. Cogn. Disord. 14, 77–83. 10.1159/000064928 12145454

[B85] Marseglia A. Fratiglioni L. Kalpouzos G. Wang R. Backman L. Xu W. (2019). Prediabetes and diabetes accelerate cognitive decline and predict microvascular lesions: a population-based cohort study. Alzheimers. Dement. 15, 25–33. 10.1016/j.jalz.2018.06.3060 30114414

[B86] Martinez-Lapiscina E. H. Galbete C. Corella D. Toledo E. Buil-Cosiales P. Salas-Salvado J.. (2014). Genotype patterns at CLU, CR1, PICALM and APOE, cognition and Mediterranean diet: the PREDIMED-NAVARRA trial. Genes Nutr. 9:393. 10.1007/s12263-014-0393-7 24643340 PMC4026432

[B87] Marzona I. O'Donnell M. Teo K. Gao P. Anderson C. Bosch J.. (2012). Increased risk of cognitive and functional decline in patients with atrial fibrillation: results of the ONTARGET and TRANSCEND studies. CMAJ 184, E329–336. 10.1503/cmaj.111173 22371515 PMC3314061

[B88] Matej R. Tesar A. Rusina R. (2019). Alzheimer's disease and other neurodegenerative dementias in comorbidity: a clinical and neuropathological overview. Clin. Biochem. 73, 26–31. 10.1016/j.clinbiochem.2019.08.005 31400306

[B89] Mauricio M. O'Hara R. Yesavage J. A. Friedman L. Kraemer H. C. Van De Water M.. (2000). A longitudinal study of apolipoprotein-E genotype and depressive symptoms in community-dwelling older adults. Am. J. Geriatr. Psychiatry. 8, 196–200. 10.1097/00019442-200008000-00003 10910416

[B90] Merlini M. Wanner D. Nitsch R. M. (2016). Tau pathology-dependent remodelling of cerebral arteries precedes Alzheimer's disease-related microvascular cerebral amyloid angiopathy. Acta Neuropathol. 131, 737–752. 10.1007/s00401-016-1560-2 26988843 PMC4835519

[B91] Miklossy J. McGeer P. L. (2016). Common mechanisms involved in Alzheimer's disease and type 2 diabetes: a key role of chronic bacterial infection and inflammation. Aging 8, 575–588. 10.18632/aging.100921 26961231 PMC4925815

[B92] Moloney A. M. Griffin R. J. Timmons S. O'Connor R. Ravid R. O'Neill C. (2010). Defects in IGF-1 receptor, insulin receptor and IRS-1/2 in Alzheimer's disease indicate possible resistance to IGF-1 and insulin signalling. Neurobiol. Aging 31, 224–243. 10.1016/j.neurobiolaging.2008.04.002 18479783

[B93] Morris M. C. Scherr P. A. Hebert L. E. Glynn R. J. Bennett D. A. Evans D. A. (2001). Association of incident Alzheimer disease and blood pressure measured from 13 years before to 2 years after diagnosis in a large community study. Arch. Neurol. 58, 1640–1646. 10.1001/archneur.58.10.1640 11594923

[B94] Mukherjee A. Morales-Scheihing D. Butler P. C. Soto C. (2015). Type 2 diabetes as a protein misfolding disease. Trends Mol. Med. 21, 439–449. 10.1016/j.molmed.2015.04.005 25998900 PMC4492843

[B95] Mullins R. J. Mustapic M. Chia C. W. Carlson O. Gulyani S. Tran J.. (2019). A pilot study of exenatide actions in Alzheimer's disease. Curr. Alzheimer Res. 16, 741–752. 10.2174/1567205016666190913155950 31518224 PMC7476877

[B96] Newcombe E. A. Camats-Perna J. Silva M. L. Valmas N. Huat T. J. Medeiros R. (2018). Inflammation: the link between comorbidities, genetics, and Alzheimer's disease. J. Neuroinflammation. 15:276. 10.1186/s12974-018-1313-3 30249283 PMC6154824

[B97] Newman A. B. Fitzpatrick A. L. Lopez O. Jackson S. Lyketsos C. Jagust W.. (2005). Dementia and Alzheimer's disease incidence in relationship to cardiovascular disease in the Cardiovascular Health Study cohort. J. Am. Geriatr. Soc. 53, 1101–1107. 10.1111/j.1532-5415.2005.53360.x 16108925

[B98] Noguchi-Shinohara M. Komatsu J. Samuraki M. Matsunari I. Ikeda T. Sakai K.. (2017). Cerebral amyloid angiopathy-related microbleeds and cerebrospinal fluid biomarkers in Alzheimer's Disease. J. Alzheimers. Dis. 55, 905–913. 10.3233/JAD-160651 27802236

[B99] Notkola I. L. Sulkava R. Pekkanen J. Erkinjuntti T. Ehnholm C. Kivinen P.. (1998). Serum total cholesterol, apolipoprotein E epsilon 4 allele, and Alzheimer's disease. Neuroepidemiology 17, 14–20. 10.1159/000026149 9549720

[B100] O'Brien R. J. Wong P. C. (2011). Amyloid precursor protein processing and Alzheimer's disease. Annu. Rev. Neurosci. 34, 185–204. 10.1146/annurev-neuro-061010-113613 21456963 PMC3174086

[B101] O'Donnell S. Borowski K. Espin-Garcia O. Milgrom R. Kabakchiev B. Stempak J.. (2019). The unsolved link of genetic markers and Crohn's disease progression: a north american cohort experience. Inflamm. Bowel Dis. 25, 1541–1549. 10.1093/ibd/izz016 30801121

[B102] Ott A. Breteler M. M. de Bruyne M. C. van Harskamp F. Grobbee D. E. Hofman A. (1997). Atrial fibrillation and dementia in a population-based study. The Rotterdam Study. Stroke 28, 316–321. 10.1161/01.STR.28.2.316 9040682

[B103] Ownby R. L. Crocco E. Acevedo A. John V. Loewenstein D. (2006). Depression and risk for Alzheimer disease: systematic review, meta-analysis, and metaregression analysis. Arch. Gen. Psychiatry. 63, 530–538. 10.1001/archpsyc.63.5.530 16651510 PMC3530614

[B104] Paresce D. M. Ghosh R. N. Maxfield F. R. (1996). Microglial cells internalize aggregates of the Alzheimer's disease amyloid beta-protein via a scavenger receptor. Neuron 17, 553–565. 10.1016/S0896-6273(00)80187-7 8816718

[B105] Parissis J. T. Adamopoulos S. Rigas A. Kostakis G. Karatzas D. Venetsanou K.. (2004). Comparison of circulating proinflammatory cytokines and soluble apoptosis mediators in patients with chronic heart failure with versus without symptoms of depression. Am. J. Cardiol. 94, 1326–1328. 10.1016/j.amjcard.2004.07.127 15541260

[B106] Peila R. Rodriguez B. L. Launer L. J. Honolulu-Asia Aging S. (2002). Type 2 diabetes, APOE gene, and the risk for dementia and related pathologies: the Honolulu-Asia Aging Study. Diabetes 51, 1256–1262. 10.2337/diabetes.51.4.1256 11916953

[B107] Peila R. White L. R. Masaki K. Petrovitch H. Launer L. J. (2006). Reducing the risk of dementia: efficacy of long-term treatment of hypertension. Stroke 37, 1165–1170. 10.1161/01.STR.0000217653.01615.93 16601212

[B108] Petrovitch H. White L. Masaki K. H. Ross G. W. Abbott R. D. Rodriguez B. L.. (1998). Influence of myocardial infarction, coronary artery bypass surgery, and stroke on cognitive impairment in late life. Am. J. Cardiol. 81, 1017–1021. 10.1016/S0002-9149(98)00082-4 9576163

[B109] Petrovitch H. White L. R. Izmirilian G. Ross G. W. Havlik R. J. Markesbery W.. (2000). Midlife blood pressure and neuritic plaques, neurofibrillary tangles, and brain weight at death: the HAAS. Honolulu-Asia aging Study. Neurobiol Aging 21, 57–62. 10.1016/S0197-4580(00)00106-8 10794849

[B110] Prins N. D. van Dijk E. J. den Heijer T. Vermeer S. E. Koudstaal P. J. Oudkerk M.. (2004). Cerebral white matter lesions and the risk of dementia. Arch. Neurol. 61, 1531–1534. 10.1001/archneur.61.10.1531 15477506

[B111] Pugazhenthi S. Qin L. Reddy P. H. (2017). Common neurodegenerative pathways in obesity, diabetes, and Alzheimer's disease. Biochim Biophys Acta Mol Basis Dis. 1863, 1037–1045. 10.1016/j.bbadis.2016.04.017 27156888 PMC5344771

[B112] Rawlings A. M. Sharrett A. R. Mosley T. H. Ballew S. H. Deal J. A. Selvin E. (2017). Glucose peaks and the risk of dementia and 20-year cognitive decline. Diabetes Care 40, 879–886. 10.2337/dc16-2203 28500217 PMC5481977

[B113] Rawlings A. M. Sharrett A. R. Schneider A. L. Coresh J. Albert M. Couper D.. (2014). Diabetes in midlife and cognitive change over 20 years: a cohort study. Ann. Intern. Med. 161, 785–793. 10.7326/M14-0737 25437406 PMC4432464

[B114] Ray M. Ruan J. Zhang W. (2008). Variations in the transcriptome of Alzheimer's disease reveal molecular networks involved in cardiovascular diseases. Genome Biol. 9:R148. 10.1186/gb-2008-9-10-r148 18842138 PMC2760875

[B115] Reger M. A. Henderson S. T. Hale C. Cholerton B. Baker L. D. Watson G. S.. (2004). Effects of beta-hydroxybutyrate on cognition in memory-impaired adults. Neurobiol. Aging 25, 311–314. 10.1016/S0197-4580(03)00087-3 15123336

[B116] Roberts R. O. Knopman D. S. Geda Y. E. Cha R. H. Roger V. L. Petersen R. C. (2010). Coronary heart disease is associated with non-amnestic mild cognitive impairment. Neurobiol. Aging 31, 1894–1902. 10.1016/j.neurobiolaging.2008.10.018 19091445 PMC2888961

[B117] Santiago J. A. Bottero V. Potashkin J. A. (2017). Dissecting the molecular mechanisms of neurodegenerative diseases through network biology. Front. Aging Neurosci. 9:166. 10.3389/fnagi.2017.00166 28611656 PMC5446999

[B118] Santiago J. A. Bottero V. Potashkin J. A. (2019). Transcriptomic and network analysis highlight the association of diabetes at different stages of Alzheimer's Disease. Front. Neurosci. 13:1273. 10.3389/fnins.2019.01273 31849586 PMC6895844

[B119] Santiago J. A. Potashkin J. A. (2014). A network approach to clinical intervention in neurodegenerative diseases. Trends Mol. Med. 20, 694–703. 10.1016/j.molmed.2014.10.002 25455073

[B120] Sluggett J. K. Koponen M. Bell J. S. Taipale H. Tanskanen A. Tiihonen J.. (2020). Metformin and risk of Alzheimer's disease among community-dwelling people with diabetes: a national case-control study. J. Clin. Endocrinol. Metab. 105:dgz234. 10.1210/clinem/dgz234 31778170

[B121] Snowdon D. A. Greiner L. H. Mortimer J. A. Riley K. P. Greiner P. A. Markesbery W. R. (1997). Brain infarction and the clinical expression of Alzheimer disease. The Nun Study. JAMA 277, 813–817. 10.1001/jama.1997.03550020046024 9052711

[B122] Speck C. E. Kukull W. A. Brenner D. E. Bowen J. D. McCormick W. C. Teri L.. (1995). History of depression as a risk factor for Alzheimer's disease. Epidemiology 6, 366–369. 10.1097/00001648-199507000-00006 7548342

[B123] Stewart R. Russ C. Richards M. Brayne C. Lovestone S. Mann A. (2001). Depression, APOE genotype and subjective memory impairment: a cross-sectional study in an African-Caribbean population. Psychol. Med. 31, 431–440. 10.1017/S0033291701003257 11305851

[B124] Suarez E. C. Lewis J. G. Krishnan R. R. Young K. H. (2004). Enhanced expression of cytokines and chemokines by blood monocytes to in vitro lipopolysaccharide stimulation are associated with hostility and severity of depressive symptoms in healthy women. Psychoneuroendocrinology 29, 1119–1128. 10.1016/j.psyneuen.2004.01.002 15219635

[B125] Sutinen E. M. Pirttila T. Anderson G. Salminen A. Ojala J. O. (2012). Proinflammatory interleukin-18 increases Alzheimer's disease-associated amyloid-beta production in human neuron-like cells. J. Neuroinflammation. 9:199. 10.1186/1742-2094-9-199 22898493 PMC3458954

[B126] Taylor M. K. Sullivan D. K. Mahnken J. D. Burns J. M. Swerdlow R. H. (2018). Feasibility and efficacy data from a ketogenic diet intervention in Alzheimer's disease. Alzheimers Dement. 4, 28–36. 10.1016/j.trci.2017.11.002 29955649 PMC6021549

[B127] Thong J. Y. Hilal S. Wang Y. Soon H. W. Dong Y. Collinson S. L.. (2013). Association of silent lacunar infarct with brain atrophy and cognitive impairment. J. Neurol. Neurosurg. Psychiatr. 84, 1219–1225. 10.1136/jnnp-2013-305310 23933740

[B128] Toledo J. B. Arnold S. E. Raible K. Brettschneider J. Xie S. X. Grossman M.. (2013). Contribution of cerebrovascular disease in autopsy confirmed neurodegenerative disease cases in the National Alzheimer's Coordinating Centre. Brain 136, 2697–2706. 10.1093/brain/awt188 23842566 PMC3858112

[B129] van den Brink A. C. Brouwer-Brolsma E. M. Berendsen A. A. M. van de Rest O. (2019). The mediterranean, dietary approaches to stop hypertension (DASH), and mediterranean-DASH intervention for neurodegenerative delay (MIND) diets are associated with less cognitive decline and a lower risk of Alzheimer's disease-a review. Adv. Nutr. 10, 1040–1065. 10.1093/advances/nmz054 31209456 PMC6855954

[B130] van der Maaden T. Hendriks S. A. de Vet H. C. Zomerhuis M. T. Smalbrugge M. Jansma E. P.. (2015). Antibiotic use and associated factors in patients with dementia: a systematic review. Drugs Aging 32, 43–56. 10.1007/s40266-014-0223-z 25385686

[B131] van Dijk E. J. Breteler M. M. Schmidt R. Berger K. Nilsson L. G. Oudkerk M.. (2004). The association between blood pressure, hypertension, and cerebral white matter lesions: cardiovascular determinants of dementia study. Hypertension 44, 625–630. 10.1161/01.HYP.0000145857.98904.20 15466662

[B132] van Oijen M. de Jong F. J. Witteman J. C. Hofman A. Koudstaal P. J. Breteler M. M. (2007). Atherosclerosis and risk for dementia. Ann. Neurol. 61, 403–410. 10.1002/ana.21073 17328068

[B133] Vermeer S. E. Prins N. D. den Heijer T. Hofman A. Koudstaal P. J. Breteler M. M. (2003). Silent brain infarcts and the risk of dementia and cognitive decline. N. Engl. J. Med. 348, 1215–1222. 10.1056/NEJMoa022066 12660385

[B134] Weggen S. Beher D. (2012). Molecular consequences of amyloid precursor protein and presenilin mutations causing autosomal-dominant Alzheimer's disease. Alzheimers. Res. Ther. 4:9. 10.1186/alzrt107 22494386 PMC3334542

[B135] Winkler E. A. Nishida Y. Sagare A. P. Rege S. V. Bell R. D. Perlmutter D.. (2015). GLUT1 reductions exacerbate Alzheimer's disease vasculo-neuronal dysfunction and degeneration. Nat. Neurosci. 18, 521–530. 10.1038/nn.3966 25730668 PMC4734893

[B136] Wolters F. J. Segufa R. A. Darweesh S. K. L. Bos D. Ikram M. A. Sabayan B.. (2018). Coronary heart disease, heart failure, and the risk of dementia: a systematic review and meta-analysis. Alzheimers. Dement. 14, 1493–1504. 10.1016/j.jalz.2018.01.007 29494808

[B137] Xue M. Xu W. Ou Y. N. Cao X. P. Tan M. S. Tan L.. (2019). Diabetes mellitus and risks of cognitive impairment and dementia: A systematic review and meta-analysis of 144 prospective studies. Ageing Res. Rev. 55:100944. 10.1016/j.arr.2019.100944 31430566

[B138] Yaffe K. Blackwell T. Whitmer R. A. Krueger K. Barrett Connor E. (2006). Glycosylated hemoglobin level and development of mild cognitive impairment or dementia in older women. J. Nutr. Health Aging. 10, 293–295. 16886099

[B139] Yarchoan M. Xie S. X. Kling M. A. Toledo J. B. Wolk D. A. Lee E. B.. (2012). Cerebrovascular atherosclerosis correlates with Alzheimer pathology in neurodegenerative dementias. Brain 135, 3749–3756. 10.1093/brain/aws271 23204143 PMC3577102

[B140] Yasar S. Corrada M. Brookmeyer R. Kawas C. (2005). Calcium channel blockers and risk of AD: the Baltimore Longitudinal Study of Aging. Neurobiol. Aging 26, 157–163. 10.1016/j.neurobiolaging.2004.03.009 15582745

[B141] Yokoyama J. S. Wang Y. Schork A. J. Thompson W. K. Karch C. M. Cruchaga C.. (2016). Association between genetic traits for immune-mediated diseases and Alzheimer Disease. JAMA Neurol. 73, 691–697. 10.1001/jamaneurol.2016.0150 27088644 PMC4905783

[B142] Zhang B. Wang H. E. Bai Y. M. Tsai S. J. Su T. P. Chen T. J.. (2020). Inflammatory bowel disease is associated with higher dementia risk: a nationwide longitudinal study. Gut 70, 85–91. 10.1136/gutjnl-2020-320789 32576641

[B143] Zhang Z. Q. Holscher C. (2020). GIP has neuroprotective effects in Alzheimer and Parkinson's disease models. Peptides 125:170184. 10.1016/j.peptides.2019.170184 31705913

[B144] Zheng F. Yan L. Yang Z. Zhong B. Xie W. (2018). HbA1c, diabetes and cognitive decline: the English Longitudinal Study of Ageing. Diabetologia 61, 839–848. 10.1007/s00125-017-4541-7 29368156 PMC6448974

[B145] Zhou J. Yu J. T. Wang H. F. Meng X. F. Tan C. C. Wang J.. (2015). Association between stroke and Alzheimer's disease: systematic review and meta-analysis. J. Alzheimers. Dis. 43, 479–489. 10.3233/JAD-140666 25096624

[B146] Zubenko G. S. Henderson R. Stiffler J. S. Stabler S. Rosen J. Kaplan B. B. (1996). Association of the APOE epsilon 4 allele with clinical subtypes of late life depression. Biol. Psychiatry 40, 1008–1016. 10.1016/S0006-3223(96)00046-7 8915560

